# C-reactive protein-to-albumin ratio in peripheral artery disease

**DOI:** 10.1515/med-2025-1280

**Published:** 2025-11-15

**Authors:** Pandit Bagus Tri Saputra, Dinda Dwi Purwati, Pratista Oktafia, Roy Bagus Kurniawan, Cornelia Ghea Savitri, Johanes Nugroho Eko Putranto, Chaq El Chaq Zamzam Multazam, Mario D’Oria, Firas Farisi Alkaff

**Affiliations:** Department of Cardiology and Vascular Medicine, Faculty of Medicine, Universitas Airlangga, Surabaya East Java, Indonesia; Department of Cardiology and Vascular Medicine, Dr. Soetomo General Academic Hospital, Surabaya East Java, Indonesia; Faculty of Medicine, Universitas Airlangga, Surabaya, East Java, Indonesia; National Heart and Lung Institute, Imperial College London, Guy Scadding Building, London SW3 6LY, United Kingdom; Division of Vascular and Endovascular Surgery, Department of Clinical Surgical and Health Sciences, University of Trieste, Trieste, Italy; Division of Nephrology, Department of Internal Medicine, University Medical Center Groningen, Hanzeplein 1, Groningen, 9713GZ, The Netherlands; Division of Pharmacology and Therapy, Department of Anatomy, Histology, and Pharmacology, Faculty of Medicine, Universitas Airlangga, Jl. Mayjen Prof. Dr. Moestopo No 47, Surabaya, East Java, Indonesia

**Keywords:** peripheral artery disease, C-reactive protein-to-albumin ratio, prognosis, mortality, amputation

## Abstract

**Aim:**

The aim of this study was to assess the prognostic performance of the C-reactive protein-to-albumin ratio (CAR) in predicting mortality and amputation of peripheral artery disease (PAD) patients undergoing endovascular therapy (EVT).

**Methods:**

We conducted a systematic search from the inception date to June 12, 2025, in eight databases and a manual search to cover gray literature. High and low CAR were defined according to the optimal cut-off from each study. Meta-analysis was performed to pool prognostic performance.

**Result:**

A total of 1,451 subjects from five observational studies were included. The prevalence of mortality and amputation was 4.7 and 21.4%, respectively. Pre-procedural high CAR was associated with a higher risk of mortality (risk ratio [RR] 3.11; 95% confidence interval [CI] 1.22–8.18; *I* ^2^ = 40%) and amputation (RR 3.62; 95% CI 1.98–6.63; *I* ^2^ = 10%). The area under the receiver operating characteristic (ROC) curve of the summary ROC curve of high CAR was 0.75 (sensitivity 77% and specificity 56%) for mortality and 0.85 (sensitivity 85% and specificity 52%) for amputation. The positive predictive value of CAR to predict mortality and amputation was 29.9 and 10.5%, respectively, while the negative predictive value was 90.5 and 97.8%, respectively.

**Conclusion:**

CAR was a potential prognostic biomarker to predict mortality and amputation in PAD patients undergoing EVT.

## Graphical abstract

**Figure:**
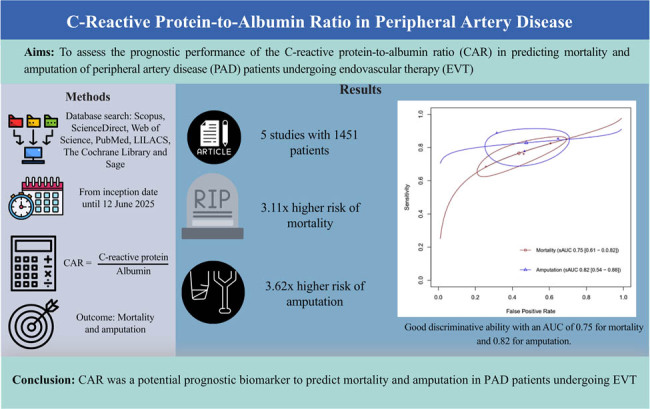

## 1 Introduction

Peripheral artery disease (PAD) refers to stenosis or occlusion of the arteries supplying the lower limbs, typically due to atherosclerosis, which is characterized by low-grade chronic inflammation [1,2]. Vascular wall inflammation is the basis of PAD pathophysiology and is associated with PAD severity [3]. Management of PAD that impairs daily activities often requires revascularization therapy, which may include endovascular therapy (EVT), open surgery, or hybrid approaches [4,5]. However, a feasible, cost-effective, and accurate prognostic biomarker has yet to be recommended.

C-reactive protein (CRP) is one of the most commonly used inflammatory biomarkers worldwide [6]. Previous studies showed the association between CRP and major cardiovascular events, including in PAD patients [7,8]. Albumin also serves as a prognostic laboratory parameter that is associated with mortality in various diseases [9]. Albumin concentration decreases as inflammation intensity increases [10]. The combination of CRP-to-albumin ratio (CAR) was reported as a better prognostic biomarker than CRP or albumin alone to predict mortality and major adverse events [11–14].

The efficacy of CAR examination holds the potential to serve as a prognostic biomarker for PAD patients undergoing EVT. The importance of simple, feasible, and low-cost prognostic biomarkers helps clinicians to identify high-risk PAD patients before the EVT procedure. However, there is no solid evidence providing accurate estimations of CAR’s prognostic performance. Therefore, this meta-analysis aimed to evaluate the prognostic roles of CAR to predict the outcomes of PAD patients undergoing EVT.

## 2 Materials and methods

The protocol of this review was registered in the International Prospective Register of Systematic Reviews (PROSPERO) under the registration number CRD42024527902 and has been written according to the guidelines from the Preferred Reporting Items for Systematic Reviews and Meta-Analysis (PRISMA) 2020 (Table S1) [14].

### 2.1 Eligibility criteria

This review includes clinical trials and observational studies. The inclusion criteria were (1) PAD patients undergoing EVT, (2) reporting the CAR, (3) reporting of primary outcomes (mortality and/or amputation above or below the ankle) and/or secondary outcomes (restenosis), and (4) written in English. Studies that did not conduct CAR analysis (either CRP only, albumin only, or CRP and albumin without ratio analysis), only available as abstracts, case reports, reviews, meta-analyses, comments, or editorials, were excluded from this study. CAR is defined as C-reactive protein (as numerator) divided by albumin serum (as denumerator) [15]. Only studies reporting a CAR examination that was performed before the angiography procedure were included.

### 2.2 Data search strategy

A systematic search from the inception date to June 12, 2025, encompassing several databases such as ProQuest, Scopus, ScienceDirect, Web of Science, PubMed, LILACS, The Cochrane Library, and Sage, was performed. Two researchers conducted independent reviews of relevant studies. Any discrepancies were resolved together among the authors. The search terms applied were (“C-Reactive protein to albumin ratio” OR “CRP to albumin ratio” OR “CRP to prealbumin ratio” OR “CRP-Albumin ratio”) AND (“Peripheral Artery Disease” OR “Chronic Limb Threatening Ischemia” OR “Chronic Limb Ischemia” OR “Peripheral vascular disease” OR “Peripheral vascular occlusive disease”). The manual search was also performed to increase the coverage of gray literature. Further details can be observed in Table S2.

### 2.3 Quality assessment and data extraction

The quality of the selected studies was analyzed using the Newcastle-Ottawa Scale (NOS) quality assessment tool for observational studies [16], which was then categorized into three groups. Studies with ≥7 points were considered “good,” those with 2–6 points were considered “fair,” and those with ≤1 point were considered “poor” quality of study [16]. Two investigators conducted bias assessment independently, and any discrepancies were discussed among authors. The relevant data from the included studies were extracted into a pre-specified table comprising the first author’s name, study design, geographical location, sample size, participant characteristics, age distribution, patient’s comorbidity, the received medication, and outcomes. Any discrepancies in the extraction process were resolved through discussion among the authors.

### 2.4 Statistical analysis

The meta-analysis was conducted using R software version 4.2.2 (Posit PBC, USA). A default random-effect model meta-analysis was used to anticipate heterogeneity in all analyses. We estimated the association of CAR and outcomes with the risk ratio (RR). Furthermore, we collected diagnostic measures such as true positive, false positive, true negative, and false negative to generate pooled sensitivity, specificity, positive predictive value (PPV), negative predictive value (NPV), and area under the ROC curve (AUC). For the generation of pooled sensitivity, specificity, PPV, and NPV, the univariate model was utilized. Additionally, the bivariate model was used to establish summary receiver operating characteristic (sROC) curves and AUC. AUCs were not directly extracted from the AUC results of each study; instead, they were analyzed and aggregated from each diagnostic measure. The AUC value was interpreted according to Jayawant et Mandrekar [17], so that an AUC value of 0.5 indicates that CAR had no ability to distinguish patients’ outcomes, while values of 0.7–0.8 were considered to indicate an acceptable diagnostic power, values of 0.8–0.9 were considered excellent, and more than 0.9 would suggest outstanding discriminatory power.

Heterogeneity levels were categorized according to Higgins’ *I*² values, classifying them as negligible (0–25%), low (25–50%), moderate (50–75%), or high (>75%) [18]. Additionally, we performed leave-one-out sensitivity analyses to assess the influence of individual studies on the pooled estimate and to provide further clarity on the observed heterogeneity.

## 3 Results

### 3.1 Study selection and quality assessment

**Figure 1:**
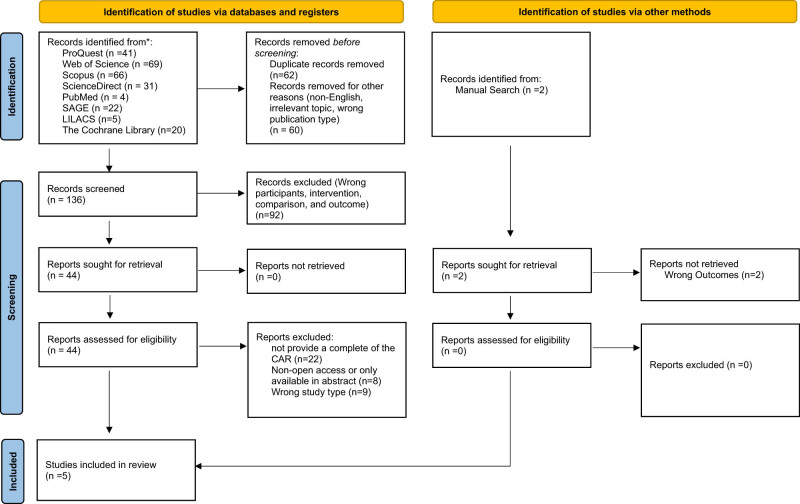
PRISMA 2020 flow diagram of study selection.

A systematic search was conducted across eight databases, resulting in a total of 258 titles and abstracts. A total of 122 titles and abstracts were excluded due to duplication, non-English language, irrelevant topics, or wrong publication type. After having screened and reviewed the full texts, five observational publications were eventually included in this meta-analysis [19–23]. The process by which studies were included in this review has been delineated within the PRISMA flow diagram (Figure 1). The risk of bias assessment showed that none of the included studies was considered high risk of bias (Tables S3 and S4).

### 3.2 Study characteristics

Out of the 1,451 total participants, 1186 (81.7%) were males, and the average age was older than 61 years. Four studies reported the severity of PAD, with the highest prevalence in Rutherford stage 3 involving 441 patients (28.2%) and Fontaine stage 2 involving 569 patients (57.1%). The comorbidities and baseline characteristics of the included studies are presented in Table 1.

**Table 1:** Characteristics of included studies

No	Author	Study design	Country	Study period	Sample size	Study population	Age (mean)	Male, *n* (%)	CAR examination	Cut-off	Comorbidities	Reported outcome (s)	Severity of PAD (*n*)
1	Kim et al. [23]	Retrospective Cohort	Korea	2009–2019	307	Patients with symptomatic lower extremity PAD who underwent EVT	68.8	225 (73.30%)	Before the EVT	0.63	HT (76.5%)	MACCE All-cause death Cardiac death Amputation Myocardial infarction Stroke MALE Repeat revascularization	N/A
							70.5				DM (53.7%)		
							70.4				Dyslipidemia (69%)		
											Smoking (32.9%)		
											CKD (29.6%)		
											ESRD (13.4%)		
											Previous MI (11.4%)		
											Previous EVT (6.5%)		
2	Süleymanoğlu et al. [19]	Cross-sectional	Turkey	2015–2020	149	Patients with the clinical features of possible PAD and/or CLTI who underwent EVT	68.49	127 (85%)	Before the EVT	0.19	HT (64.4%)	All-cause mortality Amputation MACLE	**Rutherford**
											DM (47.7%)		Stage 0: 3
											Hyperlipidemia (35.6%)		Stage 1: 6
											Smoking (53.7%)		Stage 2: 33
											CAD (66.4%)		Stage 3: 83
											CKD (22.5%)		Stage 4: 10
											AF (6%)		Stage 5: 10
											CHF (20.1%)		Stage 6: 4
3	Tasbulak et al. [22]	Retrospective Cohort	Turkey	2015–2020	685	Patients with symptomatic lower extremity PAD who underwent EVT	62.32	588 (85.83%)	Before the EVT	0.80	HT (72.8%)	Mortality Amputation Restenosis MI Stroke Coronary revascularization Hyperbaric oxygen treatment	**Fontaine**
											DM (59.7%)		Stage II: 484
											CKD (25.3%)		Stage III: 117
											COPD (16%)		Stage IV: 84
											AF (9.3%)		**Rutherford**
											CAD (60.7%)		Stage 1: 1
											Previous CVD (7.7%)		Stage 2: 120
											Smoking (52.1%)		Stage 3: 358
											Previous PAD (33.2%)		Stage 4: 107
													Stage 5: 63
													Stage 6: 36
4	Çalık et al. [20]*	Retrospective cohort	Turkey	2015–2018	138	Patients undergoing EVT for PAD	61.5	112 (81.2%)	Before the EVT	0.29	HT (77.5%)	in-stent restenosis (ISR)	**Fontaine**
											DM (55.8%)		Stage II: 85
											Dyslipidemia (73.2%)		
											Smoking (71.7%)		Stage III: 44
											CAD (65.2%)		Stage IV: 9
5	Panç et al. [21]*	Retrospective Cohort	Turkey	2015–2019	172	Patients undergoing EVT for PAD	64.1	134 (77.9%)	Before the EVT	N/A	HT (55.2%)	All-cause death Major amputation Minor amputation	**Fontaine**
											DM (88.4%)		Stage III: 61
											Hyperlipidemia (58.7%)		Stage IV: 111
											Smoking (58.7%)		**Rutherford**
											Prior CAD (60.5%)		Stage 4: 64
											CHF (17.4%)		Stage 5: 72
											CKD (26.7%)		Stage 6: 22
											AF (15.1%)		
											History stroke (13.5%)		
											Previous contralateral major amputation (4.7%)		
											Previous ipsilateral minor amputation (9.3%)		

Abbreviations: CAR, C-reactive protein-to-albumin ratio; PAD, peripheral artery disease; EVT, endovascular therapy; CLTI, critical limb ischemia; CIA, common iliac artery; EIA, external iliac artery; TASC, The TransAtlantic InterSociety Consensus; HT, hypertension; DM, diabetes mellitus; CAD, coronary artery disease; CKD, chronic kidney disease; ESRD, end-stage renal disease; AF, atrial fibrillation; MI, myocardial infarction; CHF, chronic heart failure; MACLE, major adverse cardiovascular and leg events; MACE, major adverse cardiovascular events; MALE, major adverse limb events; MACCE, major cardiac and cerebrovascular events.

*Study not included in meta-analysis.

### 3.3 CAR examination

All of the studies analyzed CAR in relation to adverse outcomes following post-EVT [19–23]. The timing of blood sample collection for CAR examination varied, namely at admission [23], or after 12 h of fasting [19]. However, all patient examination was performed before EVT during the index hospitalization [19–23].

#### 3.3.1 CAR for predicting mortality

**Figure 2:**
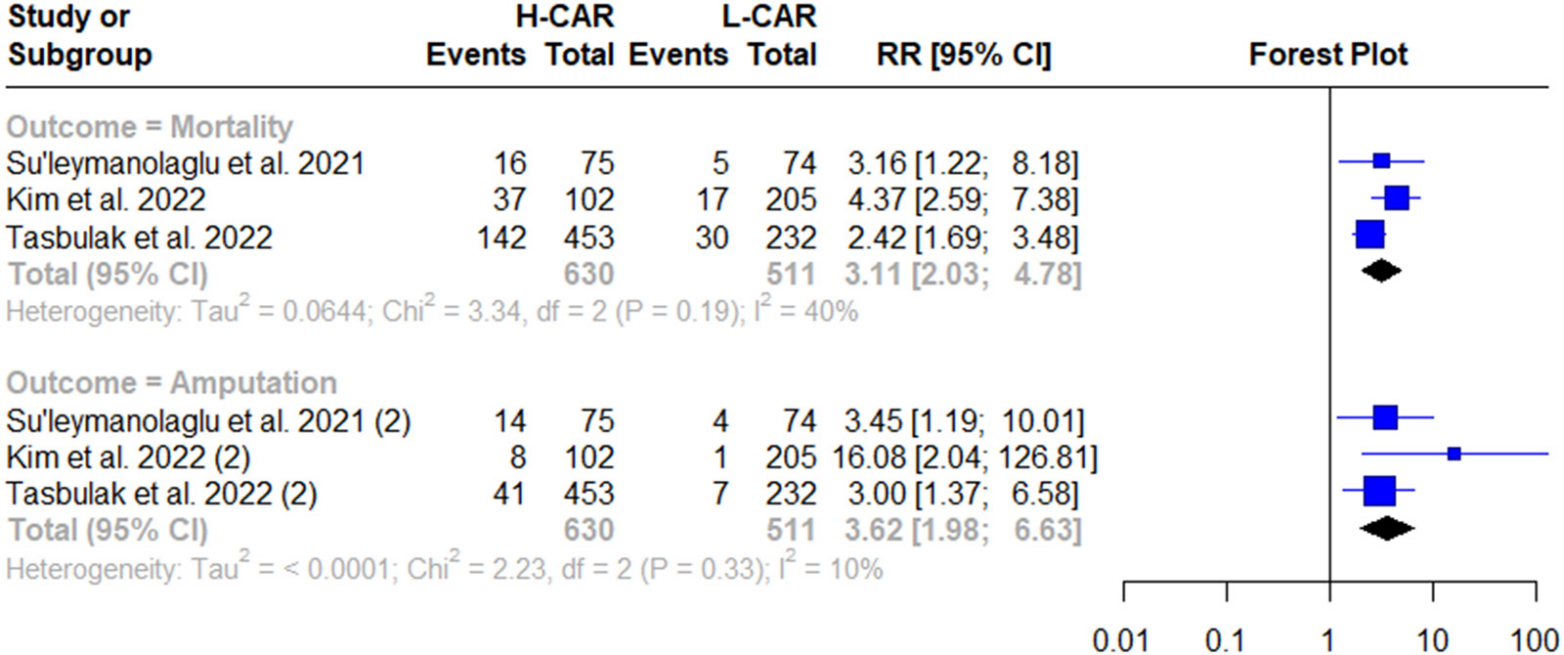
Forest plots of the association of CAR with amputation and mortality.

**Figure 3:**
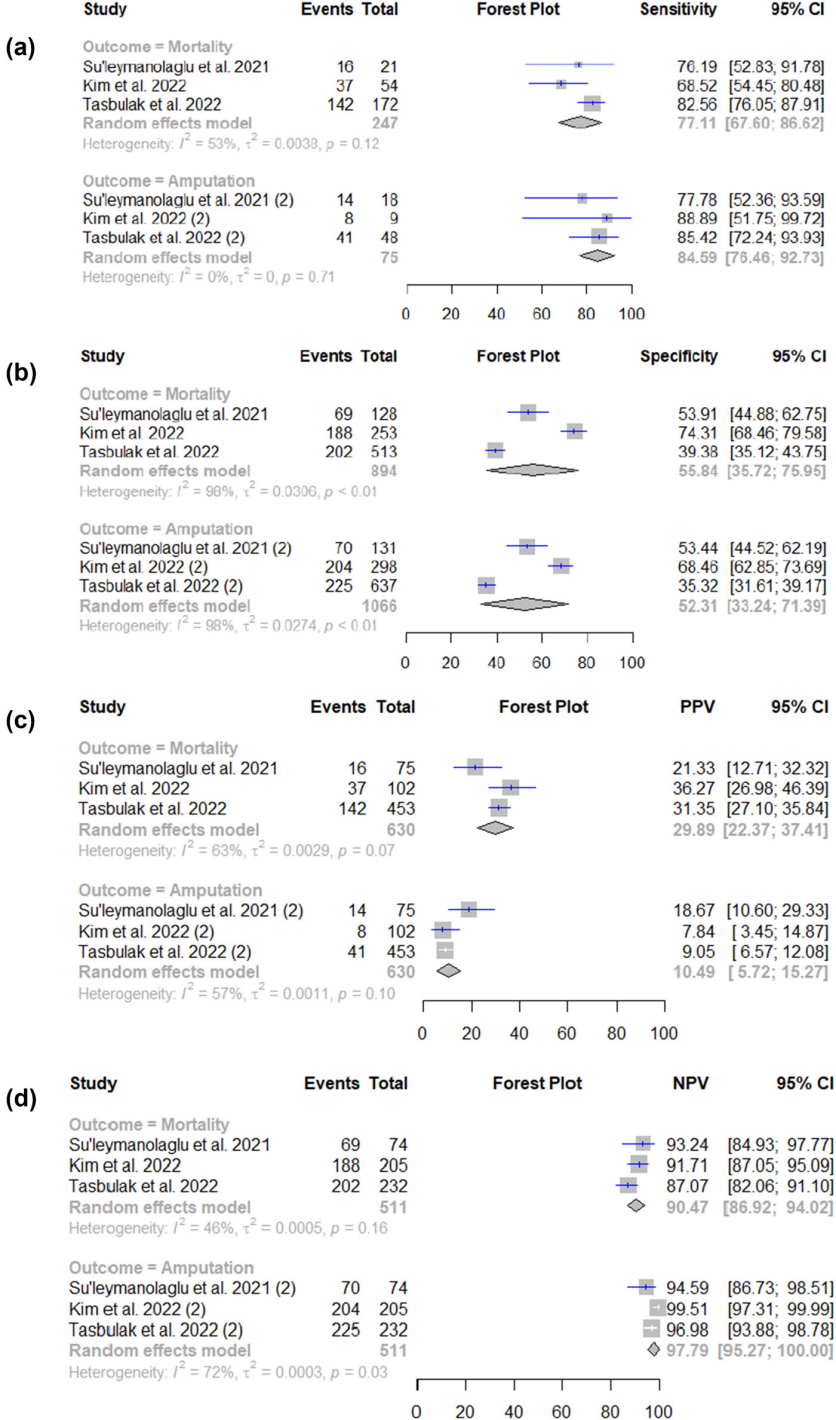
Forest plots of CAR for predicting mortality and amputation. (a) Pooled sensitivity, (b) pooled specificity, (c) pooled negative predictive value, and (d) pooled positive predictive value.

**Figure 4:**
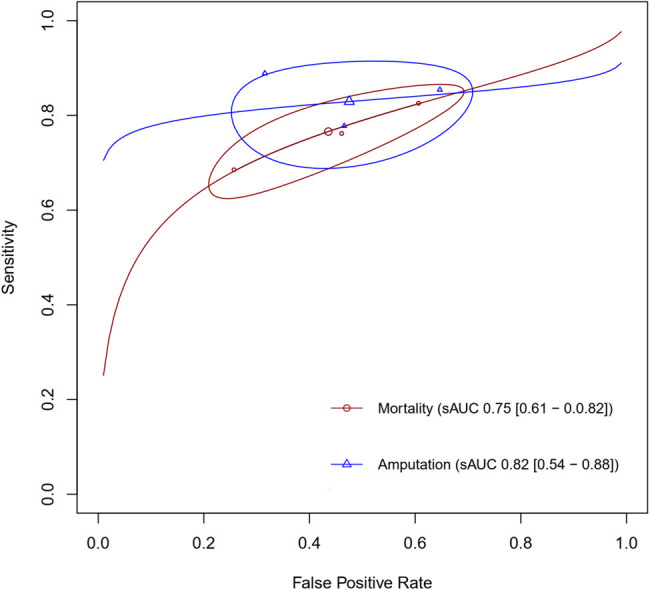
Summary ROC curves of CAR for predicting mortality and amputation.

Three studies comprising 1,141 patients were eligible for quantitative analysis. The prevalence of mortality at a range of 3-year follow-up times was 21.67% (247 patients). Comparison of mortality estimates for low and high CAR was 9.5 and 29.9%, respectively (*p* < 0.01). Patients with high CAR undergoing EVT had a 3.11 risk ratio of mortality (95% confidence interval [CI] 1.22–8.18), with an *I* ^2^ value of 40% as depicted in Figure 2. Furthermore, we conducted sensitivity, specificity, and AUC pooling for CAR in predicting mortality. The findings indicated that the sensitivity of CAR in predicting mortality was 77.11% (95% CI 67.60–86.62) and the specificity was 55.85% (95% CI 35.72–75.95). We observed significant heterogeneity among these studies from the sensitivity and specificity analysis, with *I* ^2^ = 98% and *I* ^2^ = 53%, respectively. To address this, we conducted a leave-one-out sensitivity analysis (Figures S1 and S2) on the studies assessing mortality, revealing that the omission of the study conducted by Tasbulak et al. [22] contributed to a better understanding of the observed heterogeneity [22]. The PPV and NPV were 29.89% (95% CI 22.37–37.41; *I* ^2^ = 63%) and 90.47% (95% CI 86.92–94.02; *I* ^2^ = 46%), respectively (Figure 3; Table 2). Additionally, a summary ROC curve was generated, with an AUC of 0.75 (Figure 4).

**Table 2:** The overview of CAR as a prognostic tool for predicting mortality and amputation

	Mortality		Amputation	
	Value (%)	95% CI	Value (%)	95% CI
Prevalence of H-CAR	29.89	22.37–37.41	10.49	5.72–15.27
Prevalence of L-CAR	9.53	5.98–13.08	2.21	0.00–4.73
Sensitivity	77.11	67.60–86.62	84.59	76.46–92.73
Specificity	55.84	35.72–75.95	52.31	33.24–71.39
PPV	29.89	22.37–37.41	10.49	5.72–15.27
NPV	90.47	86.92–94.02	97.79	95.27–100.00
AUC	0.75	0.60–0.81	0.85	0.53–0.88

#### 3.3.2 CAR for predicting amputation

Out of 1,586 patients, 75 (4.72%) underwent amputation at a range of 3-year follow-up times. For the comparison, the incidence of amputation was 10.49% in high CAR and 2.21% in low CAR (*p* < 0.01). The meta-analysis revealed that patients with high CAR had a 3.6 risk for amputation (RR 3.62 [95% CI 1.98–6.63]), with an *I* ^2^ value of 10% (Figure 2). In addition, the pooled sensitivity of CAR in predicting amputation was 84.59% (95% CI 76.46–92.37%; *I* ^2^ = 0%) and the specificity was 52.31% (95% CI 33.24–71.39%; *I* ^2^ = 98%). The PPV and NPV were 10.49% (95% CI 5.72–15.27) and 97.79% (95% CI 95.27–100.00), respectively. Furthermore, an sROC curve was generated, showing an sAUC value of 0.82 (95% CI 0.53–0.88) (Figure 4). The significant heterogeneity was observed in pooled specificity (*I* ^2^ = 98%, Figure 3), warranting further investigation. A leave-one-out sensitivity analysis was performed (Figure S3); yet, it resulted in an insignificant reduction of heterogeneity.

#### 3.3.3 Systematic review of secondary outcomes

The results of the study by Tasbulak et al. [22] and Kim et al. [23] showed no significant difference between high and low CAR with respect to myocardial infarction (MI) (*p* = 0.169, *p* = 0.133) and stroke (*p* = 0.169, *p* = 0.773) [22,23]. In addition, the study conducted by Çalik et al. [20] showed that a CAR value of >0.29 was found to be the optimal value to predict in-stent restenosis (ISR) (sensitivity 97.5% and specificity 88.8%, AUC 0.94; *p* < 0.01) [20]. Also, CAR was independently associated with ISR (hazards ratio [HR] 2.66 [95% CI 1.66–4.25] *p* < 0.01) [20]. Similarly, Tasbulak et al. [22] reported that patients with high CAR values had higher rates of restenosis (30.2 vs 10.3%, *p* < 0.05) [22]. After adjusting for confounding factors, the study found that high CAR values were independent predictors of restenosis in superficial femoral artery lesions (odds ratio 3.673 [95% CI 2.280–5.919]; *p* < 0.001) [22].

## 4 Discussion

PAD is a global public health issue with poor prognosis, necessitating cost-effective interventions and management of modifiable risk factors [24]. While the previous ESC-ESVS guidelines (published in 2017) [4] for PAD were lacking specific mention regarding the roles of prognostic biomarkers in this disease, the latest guideline stated that increased levels of CRP, D-dimer, fibrinogen, and NT-proBNP were associated with mortality and MACE in PAD patients [25]. This meta-analysis showed that high CAR was associated with a 3.1-fold higher risk of mortality and a 3.6-fold higher risk of amputation compared to low CAR levels in PAD patients undergoing EVT. Although direct cross-comparison remains difficult, these statistical values were considered higher than previously known laboratory prognostic biomarkers for PAD patients, such as D-dimer (RR: 2.22), hs-cTnT (RR: 3.1), adiponectin (RR: 1.99), and fibrinogen (RR: 2.08) [26–28]. Compared to CAR, high-sensitive CRP had a slightly higher RR (3.49) to predict mortality among PAD patients [29]. As noted, the included studies in this meta-analysis used CRP (not hs-CRP) [19–23], which may be more feasible and more widely used worldwide [30], especially in developing countries. Due to its capability to detect very low concentrations, the hs-CRP examination is well known to be a prognostic biomarker in various cardiovascular diseases, while CRP is more specifically used to diagnose infectious diseases [31]. However, our findings emphasize that when CRP was combined with albumin, it had a comparable association to hs-CRP in terms of mortality. Surprisingly, CAR had almost a twofold higher association with MALE (RR: 3.6) compared to hs-CRP (RR: 1.8).

This meta-analysis showed that the prognostic performance of CAR to predict mortality and amputation was satisfactory, with AUC values of 0.75 and 0.85, respectively. In addition, CAR was found to be a more accurate prognostic marker than CRP and albumin alone in predicting more severe and complex lesions in PAD patients (AUC: 0.649 vs 0.635 vs 0.632, respectively) [32]. Although the AUC value of CAR to predict mortality was considered good, this was mainly contributed by its high sensitivity (77 and 84%), while its specificity (55 and 52%) was considered moderate. In addition, CAR performance was still better than a prediction model created by Sprengers et al. [33] with 0.76 AUC (95% CI 0.71–0.80) and sensitivity at 1 and 5 years of follow-up that were 54 and 44%, respectively [33].

Our meta-analysis showed that CAR had a low PPV to predict (29%) mortality and amputation (10%) among PAD patients undergoing EVT. However, its NPV was considered high with 90% for mortality and 98% for amputation, which means that 98% of patients with low CAR had no mortality or amputation events during the 3-year follow-up. This result supports the role of CAR examination before EVT to identify patients who have more benefits from EVT. As noted, the incidence of mortality and amputation in this meta-analysis was 21 and 4.7%, respectively. The distinct outcomes incidence among diverse populations may result in different PPV and NPV [26].

Two studies showed that high CAR values were found to be associated with ISR, with an AUC value of 0.94 [20,22]. One of the most prominent factors associated with ISR was inflammation and endothelial dysfunction [20,22]. A study by Bleda et al. [34] showed that basal levels of inflammatory markers were related to an elevated number of early re-interventions following EVT for PAD [34]. After excluding patients with infectious or other inflammatory conditions, Süleymanoğlu et al. [19] reported that the inflammatory state reflected by CAR levels was strongly associated with mortality and amputation in PAD patients undergoing EVT [19]. Patients with high inflammatory status, showing elevated CAR, may require aggressive management including lipid-lowering treatment, antithrombotic therapy, anti-inflammatory drugs, exercise, and cardiovascular risk modification [35,36].

In addition, high CAR was not associated with MI and stroke after EVT [22,23]. However, it is important to note that the inflammatory process plays a significant role in the development and progression of atherosclerosis, leading to MI and stroke [37]. Further investigation is warranted. The varying baseline clinical severity and comorbidities of the study population potentially contribute to the bias of high CAR association with patient outcomes. Nevertheless, after multivariate logistic regression analysis for adjusting cofounding factor, two studies reported that CAR was still an independent predictor of amputation and mortality (age, gender, hypertension, diabetes mellitus, hyperlipidemia, chronic heart failure, smoking, CKD, atrial fibrillation, sepsis, Fontaine class, Rutherford class, Ankle-Brachial index, walking distance, hemoglobin, and The TransAtlantic InterSociety Consensus) (HR: 1.25–1.58) [19–21,23].

This meta-analysis had some limitations. First, the included studies were mostly from a Turkish population. Thus, the generalizability of this meta-analysis should be taken carefully when applied to other populations. Second, as the included studies assessed outcomes during a 3-year follow-up, the result of longer follow-up time (such as 5–10 years) may demonstrate a distinct pattern, and further studies are still warranted to confirm. Third, the included studies used different CAR cut-offs, though they reported a cut-off of ≥0.19. The number of included studies was limited regarding this specific topic; therefore, further subgroup analysis could not be done to elucidate the persisted heterogeneities. As noted, we had tried to anticipate this by performing a systematic search in eight large databases with an additional manual reference search. Therefore, further multi-center studies with larger sample sizes and longer follow-up durations are still warranted to support and confirm these present findings.

On the other hand, the strengths of this study are notable. It represents the first systematic review and meta-analysis addressing the prognostic roles of CAR in PAD patients undergoing EVT. This is particularly timely given the current intensive research on CAR’s role in atherosclerosis diseases, thereby contributing significantly to the field’s knowledge base. In addition, CAR is a simple and feasible laboratory parameter, and each component parameter is actually routinely used worldwide. The meta-analysis also poses a comprehensive approach that spans from pooled risk assessments of mortality and amputation to detailed prognostic performance metrics, such as sensitivity, specificity, NPV, PPV, and summary ROC curve analysis. We also discuss some outcomes that could not be meta-analyzed, including restenosis and MI.

## 5 Conclusions

The CAR provided good prognostic performance to predict mortality, amputation, and ISR among PAD patients undergoing EVT. The NPV of CAR to rule out mortality and amputation was 90 and 98%, respectively, during 3 years of follow-up. Therefore, CAR is a potential prognostic biomarker to predict catastrophic events in PAD patients scheduled for EVT.

## Abbreviations

ABI: Ankle-Brachial index
AF: Atrial fibrillation
AUC: Area under the ROC curve
CAD: Coronary artery disease
CAR: C-reactive protein-to-albumin ratio
CHF: Chronic heart failure
CIA: Common iliac artery
CKD: Chronic kidney disease
CLTI: Critical limb ischemia
CRP: C-reactive protein
DM: Diabetes mellitus
EIA: External iliac artery
ESRD: End-stage renal disease
EVT: Endovascular therapy
hs-CRP: High sensitive C-reactive protein
HT: Hypertension
ISR: In-stent restenosis
MACE: Major adverse cardiovascular events
MACLE: Major adverse cardiovascular and leg events
MALE: Major adverse limb events
MI: Myocardial infarction
NOS: Newcastle–Ottawa scale
NPV: Negative predictive value
PAD: Peripheral artery disease
PPV: Positive predictive value
PRISMA: Preferred Reporting Items for Systematic Reviews and Meta-analysis
PROSPERO: Prospective Register of Systematic Reviews
ROC: Receiver operating characteristic
RR: Risk ratio
sAUC: summary area under the ROC curve
sROC: summary receiver operating characteristic
TASC: The TransAtlantic InterSociety Consensus

## Supplementary Material

Supplementary material
